# Inhibition of Lon blocks cell proliferation, enhances chemosensitivity by promoting apoptosis and decreases cellular bioenergetics of bladder cancer: potential roles of Lon as a prognostic marker and therapeutic target in baldder cancer

**DOI:** 10.18632/oncotarget.2026

**Published:** 2014-05-28

**Authors:** Yongzhang Liu, Linhua Lan, Kate Huang, Rongrong Wang, Cuicui Xu, Yang Shi, Xiaoyi Wu, Zhi Wu, Jiliang Zhang, Lin Chen, Lu Wang, Xiaomin Yu, Haibo Zhu, Bin Lu

**Affiliations:** ^1^ Protein Quality Control and Diseases Laboratory, Attardi Institute of Mitochondrial Biomedicine, School of Life Sciences, Wenzhou Medical University, Wenzhou, Zhejiang, China; ^2^ Department of Pathology, The First Affiliated Hospital of Wenzhou Medical University, Wenzhou, Zhejiang, China; ^3^ Department of Urology, The Second Affiliated Hospital of Wenzhou Medical University, Wenzhou, Zhejiang, China

**Keywords:** mitochondria, protein quality control, Lon protease, bladder cancer, chemosensitivity, cellular bioenergetics

## Abstract

ATP-dependent Lon protease within mitochondrial matrix contributes to the degradation of abnormal proteins. The oxidative or hypoxic stress which represents the stress phenotype of cancer leads to up-regulation of Lon. However, the role of Lon in bladder cancer remains undefined. Here, we found that Lon expression in bladder cancer tissues was significantly higher than those in noncancerous tissues; down-regulation of Lon in bladder cancer cells significantly blocked cancer cell proliferation via suppression c-Jun N-terminal kinase (JNK) phosphorylation due to decreased reactive oxygen species (ROS) production and enhanced the sensitivity of bladder cancer cells to chemotherapeutic agents by promoting apoptosis. We further found that Lon down-regulation in bladder cancer cells decreased cellular bioenergetics as determined by measuring aerobic respiration and glycolysis using extracellular flux analyzer. The tissue microarray (TMA) results showed that high expression of Lon was related to the T and TNM stage, as well as histological grade of bladder cancer patients. We also demonstrated that Lon was an independent prognostic factor for overall survival of bladder cancer. Taken together, our data suggest that Lon could serve as a potential diagnostic biomarker and therapeutic target for treatment of bladder cancer, as well as for prediction of the effectiveness of chemotherapy.

## INTRODUCTION

Mitochondria play a primary role in cellular bioenergetics in most eukaryotic cells, which are responsible for producing nearly 95% of cellular ATP through mitochondrial oxidative phosphorylation as well as the control of cell death or survival. Protein homeostasis within mitochondria matrix is essential for the tumor viability, which is controlled precisely by the protein quality control systems including three highly evolutionarily conserved ATP-dependent proteases. As one of the three ATP-dependent serine protease located in mitochondrial matrix, Lon contributes to the degradation of abnormal proteins including misfolded, misassembled, aggregated or damaged proteins, as well as the maintenance of mitochondrial genome (mtDNA) [1, 2]. Bladder cancer is the most common urinary system malignancy, and considered as the fifth most popular neoplasm in industrialized countries [3]. Direct cystoscopic visualization of the bladder is still the gold standard for patients with microscopic or gross hematuria [4]. A high recurrence rate and the risk of progression of bladder cancer leads to the need for a frequent and long time surveillance, causing bladder cancer being the most expensive cancer to treat [5, 6]. Multiple genetic and epigenetic factors are believed to contribute to the risk of developing bladder cancer. Cumulative increase in these genetic defects also provides prognostic assessment for disease outcome. However, it is necessary to understand the molecular mechanism of bladder carcinogenesis for the development and implementation of urinary biomarkers for both diagnosis and surveillance, which will finally reduce the need for frequent cytoscopies and the cost for the treatment [7]. Moreover, the proteins found correlated with the tumor progression will also be potential targets for therapy of bladder cancer and improve the prognosis.

The involvement of mitochondria in cell death implies its critical role in probing the cellular sensitivity to anticancer drugs. Lon is an ATP-dependent serine protease with multi-functional enzyme and highly conserved from bacteria to mammalian mitochondria and peroxisomes [8, 9]. Although the majority of Lon is soluble within the mitochondrial matrix, it is also found in mitochondrial nucleoids with the roles in mtDNA maintenance [10, 11]. Lon is a nuclear DNA-encoded mitochondrial protein which exerts multiple functions in the organelle [12]. Lon can regulate the replication of mtDNA, due to its ability to bind DNA. Mammalian Lon protease binds single stranded DNA (ssDNA) with specificity for a G-rich consensus sequence either *in vitro* or *in vivo* [11, 12, 13]. In humans, Lon protease plays a crucial role in the quality control of mitochondrial proteins in the matrix by selectively degrading misfolded, unassembled or oxidatively damaged proteins and certain short-lived regulatory proteins [14, 15, 16]. Lon is a stress protein and can be induced by a number of stresses such as accumulation of unfolded proteins in endoplasmic reticulum (ER), hypoxia and other stress conditions [17, 18, 19, 20]. The Lon up-regulation may be critical for cancer cell survival by preventing abnormal mitochondrial proteins accumulation and aggregation in response to oxidative, hypoxic, and ER stress.

The maintenance of Lon homeostasis is important to cell fate, since its down-regulation leads to decreased cell proliferation and apoptosis [21, 22, 23, 24]. Mitochondria are contributed to major reactive oxygen species (ROS) generation resulted from the “electron leak” form electron transport chain (ETC) [25]. Although physiological levels of ROS play an important role in normal cell proliferation and regulating the cellular signaling, while the excessive amount of ROS production released from mitochondria leads to the activation of mitogen-activated protein kinase (MAPK) cascade including the phosphorylation of JNK, p38 and ERK [26]. Thus, ROS are involved in tumor initiation, progression, as well as maintenance.

It has been reported that over-expression and increased of proteolytic activity of Lon protease result in the enhancement of mitochondrial biogenesis and cell tumorigenesis [27, 28]. However, no report has been described how up-regulated Lon promotes bladder cancer cell survival and tumorigenesis. Therefore, a better understanding of the molecular mechanisms underlying the relationship between tumor cells of bladder and Lon will facilitate the approaches in cancer treatment.

In this study, we analyzed the mRNA and protein expression level of Lon in paired human bladder cancer tissues and adjacent normal bladder tissues by quantitative real-time PCR (qRT-PCR) and Western blot, and found that both Lon mRNA and protein expression levels in bladder cancer tissues are dramatically increased. To investigate the mechanism and biological functions of Lon involved in bladder tumorigenesis, we down-regulated Lon protein levels by using a small interfering RNA (siRNA) transfected in human bladder cancer ScaBER and UM-UC-3 cells. Our results showed that depletion of Lon in ScaBER and UM-UC-3 bladder cancer cells reduced cell proliferation and cellular bioenergetics. Moreover, we found that inhibition of Lon decreases efficacy of chemotherapeutic reagents and reduces ROS production, which activates MAPK pathway to promote tumor progression. To further understand the clinical significance of Lon protein expression in bladder cancer progression, we employed tissue microarray (TMA) to examine the expression patterns of Lon in a large cohort of bladder cancer patients' specimen and analyzed the relationship between Lon expression and clinicopathological features of bladder cancer. We found that Lon expression positively correlates to tumor grade, T stage and TNM stage. Furthermore, multivariate survival analysis result indicated that Lon protein expression is an independent prognostic factor for predicting the outcome of bladder cancer patients. These findings suggested that Lon could be used as potential clinical diagnostic and/or prognostic marker, as well as a novel target for therapy of bladder cancer patients and in predicting the effectiveness of chemotherapy.

## RESULTS

### Expression of Lon in human bladder cancer cell lines and tumor tissues

**Figure 1:**
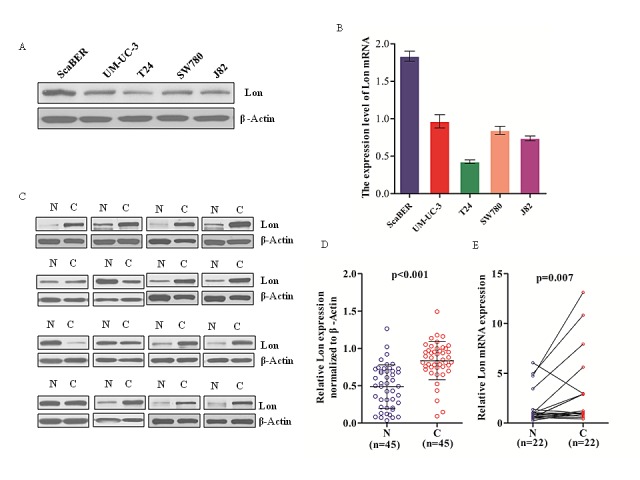
Lon expression in various human bladder cancer cell lines and 45 pairs of bladder cancer tissues and matched normal tissues (A & B) Western blotting and real-time qPCR analysis of Lon protein expression (A) and Lon mRNA (B) in bladder cancer cell lines. (C) Representative western blot are shown Lon protein expression in bladder normal (N) and matched cancer tissue (C) (n=45). (D) Relative Lon protein expression of western blotting analyses were quantified by using image J and normalized to internal control β-actin (n=45, p<0.001). (E) Lon mRNA in bladder normal (N) and cancer tissues (C) were examined by real-time qPCR (n=22, p=0.007). The expression levels of Lon protein and mRNA were shown as mean ± SD.

To understand the expression profile of Lon in human bladder cancer cell lines, we determined both the mRNA and protein expression levels of Lon in five human bladder cancer cell lines ScaBER, UM-UC-3, SW780, J82 and T24 (Figure 1A and 1B). To further validate whether Lon expression is associated with tumor progression in bladder cancer, western blot analysis was performed for 45 patients (including 45 tumor tissue and 45 matched adjacent normal tissues) diagnosed with bladder cancer. As shown in Figure 1C and Figure 1D (n=45, p<0.001), the Lon protein expression was very low but detectable in most normal bladder tissues. By contrast, Lon protein expression was markedly increased in tumor tissues (Figure 1C and 1D). In addition, the mRNA level of Lon was also significantly increased in bladder cancer tissues compared with matched normal tissues (Figure 1E, n=22, p=0.007).

### Lon depletion inhibits bladder cancer cell proliferation

**Figure 2:**
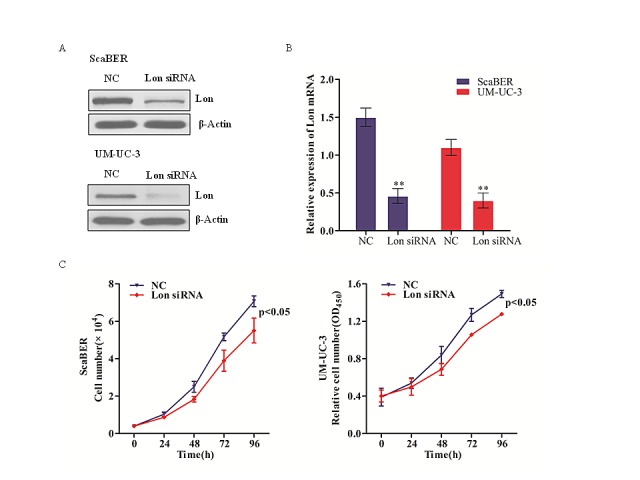
Lon depletion inhibited bladder cancer cell proliferation (A&B) RNAi-mediated knock-down of Lon in ScaBER and UM-UC-3 cells were evaluated by western blot for the protein level (A) and by real-time qPCR for the mRNA expression (B). The data shown represent results from three independent experiments. (C) The cell growth curve of ScaBER and UM-UC-3 cells transfected with control siRNA and Lon siRNA. The data are presented as mean ± SD (n=3), **p<0.01.

Uncontrolled cell proliferation is one of the most important characteristics of tumor development. To further investigate the role of Lon in bladder cancer cell proliferation, we depleted Lon with siRNA specifically targeting Lon in ScaBER and UM-UC-3 cells (Figure 2A and 2B), then assessed cellular proliferation of Lon depleted ScaBER and UM-UC-3 cells. Lon knockdown significantly decreased the proliferation of human bladder cancer cell line ScaBER (p< 0.05; Figure 2C) and UM-UC-3 (p< 0.05; Figure 2D). These data indicate that down-regulation Lon protease expression inhibits bladder cancer cell proliferation.

### Lon depletion enhances chemosensitivity of bladder cancer cells by facilitating caspase-dependent apoptosis

**Figure 3:**
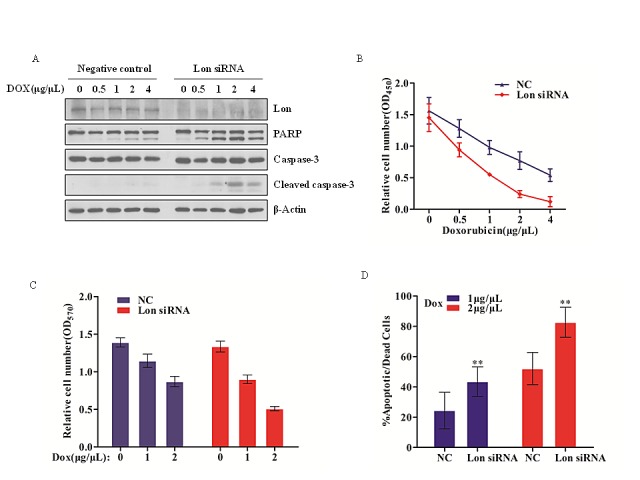
Lon depletion enhances chemosensitivity of bladder cancer cells by facilitating caspase-dependent apoptosis (A) UM-UC-3 cells were transfected with control (NC) and Lon siRNA; 48 h post transfection, cells were seeded into six-well plate followed by treatment with increasing doxorubicin as indicated for 12 h. The whole cell extracts were prepared and equal amount of protein (20 μg) was resolved on 10% SDS-PAGE and immunoblotted with indicated antibodies; β-Actin was used as a loading control. (B) 48 h post transfection, the UM-UC-3 cells transfected with control (NC) and Lon siRNA were seeded into 96-well plate, and the cells were treated with indicated concentration of doxorubicin for 12 h. MTT assay was performed to determine the cell viability as described in materials and methods. (C&D) Based on the MTT assay results, relative cell numbers after doxorubicin (0, 1, and 2 μg/μL) treatment were showed as mean ± SD of 3 replicates. (C) and the mortality rate were also analyzed (D). The data are presented as mean ± SD (n=6), **p<0.01.

Previous reports have shown that up-regulation of Lon protease in oral squamous cell carcinoma, non-small cell lung carcinoma cell line as well as 293 cells leads to mitochondrial complex I mediated ROS generation, which plays critical roles in tumorigenesis [24]. Transiently knockdown Lon in the WI-38 VA-13 human lung fibroblasts results in caspase 3 activation and promoting cell death [19]. Our previous work demonstrated that Lon knockdown leads to lymphoma cell death and Lon inhibition plays an important role in 2-cyano-3, 12-dioxooleana-1, 9-dien-28-oic acid (CDDO)-induced lymphoma cell death [22]. Based on these findings and taking into account the role of Lon in mitochondrial proteins homeostasis, we asked whether Lon over-expression in human bladder cancer cells participates in chemotherapeutic drug resistance by decreasing efficacy of cytotoxic regents to promote bladder cancer progression. To validate this hypothesis, we examined the effect of a concentration gradient of doxorubicin to control and Lon knockdown UM-UC-3 cells for 12 h, and we found a significant dose-dependent increase in apoptosis compared with negative control cells (Figure 3A). Lon down-regulated UM-UC-3 cells exhibited more cleavage of PARP and caspase-3 compared with that in negative control cells. In addition, we performed MTT assay to further investigate the Lon knockdown UM-UC-3 cell viability after doxorubicin treatment, and we found that the relative cell number was dramatically decreased in Lon down-regulated UM-UC-3 cells (Figure 3B). Moreover, we analyzed the cell survival rate difference after doxorubicin treatment between negative control cells and Lon knockdown cells, and our data showed that there is a significant increase of mortality rate in Lon down-regulated cells (Figure 3C and 3D). Taken together, these data indicate that Lon may play a critical role in anticancer drug resistance and provide a novel target for bladder cancer therapy.

### Down-regulation of Lon decreased mitochondrial ROS production as well as c-Jun N-terminal kinases (JNK) activation

**Figure 4:**
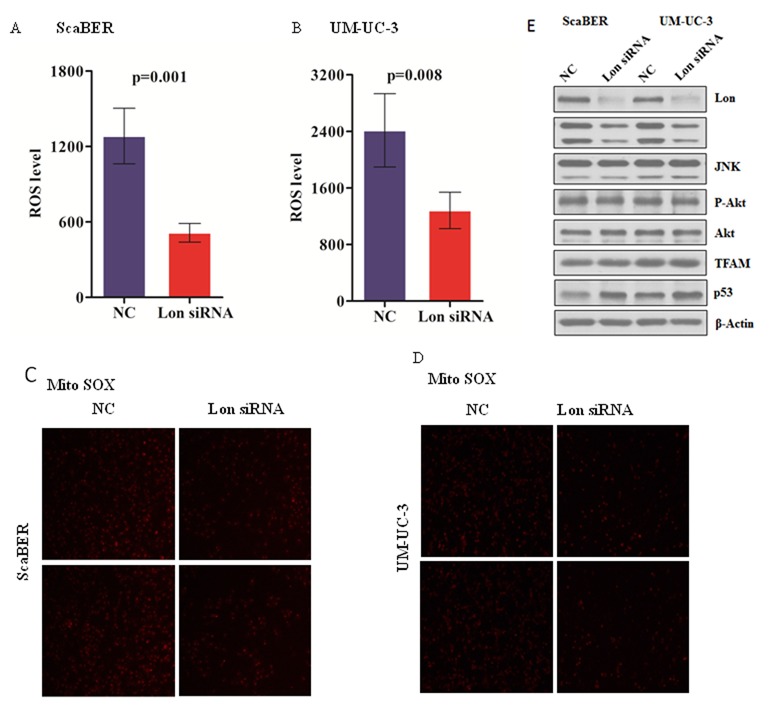
Down-regulation of Lon decreased mitochondrial ROS production as well as c-Jun N-terminal kinases (JNK) activation (A & B) Intracellular ROS of control (NC) and Lon knockdown ScaBER (A) and UM-UC-3 (B) cells was measured by using the fluorescence probe DCFH-DA. (C & D) Mitochondrial ROS level in control (NC) and Lon knockdown ScaBER (C) and UM-UC-3 (D) cells was determined using MitoSOX^TM^ Red regent. The images were taken with fluorescence microscope and representative images were shown. (E) Whole cell extracts were prepared form control (NC) and Lon knockdown ScaBER and UM-UC-3 cells. Equal amount of extracts (20 μg) were resolved on 10% SDS-PAGE and immunoblotted with indicated antibodies.

Increased ROS can stimulate Ras activity and phosphorylate MEK1/2 and ERK1/2, which are ROS level dependent tumor-associated pathways. To investigate the association of Lon expression and ROS production, DCFH-DA was used to determine the intracellular ROS production, and we found that ROS are dramatically decreased in Lon knockdown bladder cancer cells UM-UC-3 and ScaBER compared with that in control cells with regular Lon protein level (Figure 4A and 4B). Mitochondria are the major sources of ROS; we stained Lon knockdown and control cells with a mitochondrial superoxide indicator MitoSox to further confirm the alteration of mitochondrial ROS levels. We found that down-regulation of Lon significantly inhibits ROS generation in the mitochondria of human bladder cancer cells UM-UC-3 and ScaBER (Figure 4C and 4D). Thus, we examined the phosphorylation of JNK and AKT to confirm whether the downstream kinases of MAPK signaling pathway was inhibited in Lon knockdown bladder cancer cells due to suppression of ROS production. Our data showed that p-JNK was down-regulated in bladder cancer cells depleted Lon protease, while p-AKT remains unchanged (Figure 4E). Moreover, p53 was also up-regulated in response to Lon depletion in bladder cancer cells (Figure 4E), which further support our results that Lon knockdown enhances doxorubicin induced apoptosis (Figure 3A). Finally, we did not find any difference in mitochondrial transcription factor A (TFAM) level in Lon knockdown and control bladder cancer cells and which is in agreement with our previous results [29]. Collectively, our findings suggest that inhibition of Lon reduces the generation of ROS in bladder cancer cells, and may cause the inhibition of bladder cancer cell proliferation through down-regulation of JNK phosphorylation in ROS-induced MAPK pathway.

### Inhibition of mitochondrial respiration in Lon knockdown bladder cancer cells

Lon, as one of the mitochondrial matrix proteases, plays a critical role in protein quality control and appears to regulate aerobic respiratory function by its multiple functions. Under hypoxic conditions, Lon is up-regulated by the hypoxia inducible factor-1α (HIF-1α) to maintain mitochondrial protein homeostasis in a hypoxic environment. Recent studies have demonstrated that up-regulation of Lon expression may be critical for cancer cell survival by regulating stress responses induced by oxidative and hypoxic condition [28], which are common tumor-microenvironment characters of cancer cells. In addition, in view of its chaperone properties and the roles in mtDNA maintenance [29], Lon enables the mitochondria to function more efficiently in oxidative phosphorylation in response to the limited oxygen. So we speculated that Lon knockdown will inhibit cancer cell survival via reducing cellular bioenergetics of bladder cancer cells.

**Figure 5:**
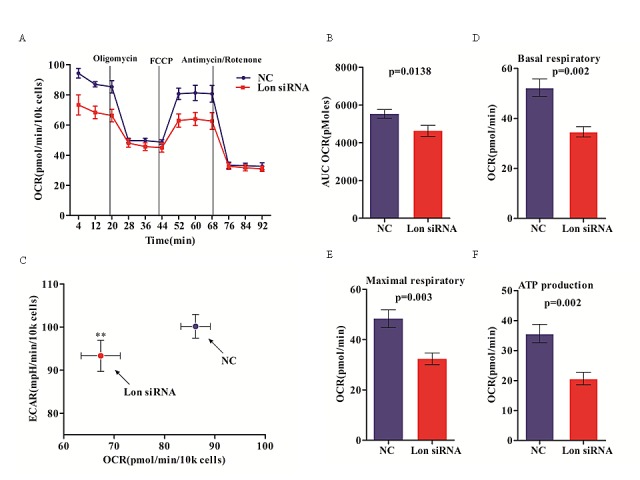
Inhibition of mitochondrial respiration in Lon knockdown bladder cancer cells (A) Mitochondrial respiration profile of UM-UC-3 cells transfected with control (NC) and Lon siRNA. The intact cellular oxygen consumption rate (OCR) and extracellular acidification rate (ECAR) were measured in real time using the Seahorse XF96 Extracellular Flux Analyzer. Basal OCR were measured at three time points, followed by sequential injection of the ATP synthase inhibitor oligomycin (1μM), the uncoupler carbonylcyanide p-trifluoromethoxyphenylhydrazone (FCCP) (1μM), the complex I inhibitor rotenone (1μM) and complex III inhibitor antimycin A(1μM). The representative graph represents the mean OCR ± SD of six replicates. (B) The area under the curve (AUC) of overall OCR of UM-UC-3 control (NC) and Lon siRNA cells was shown as AUC OCR. The data are presented as mean ± SD (n=3, p=0.0138). (C) Basal OCR and ECAR of UM-UC-3 cells transfected with control (NC) and Lon siRNA. The data are presented as mean ± SD (n=3), **p<0.01. (D) Basal OCR of the two groups of cells was calculated by the difference between baseline (time point 1-3) and oligomycin treatment (time point 4-6). The data are presented as mean ± SD (n=3, p=0.002). (E) The maximal OCR of the two groups of cells was calculated by the OCR of FCCP treatment minus antimycin A/rotenone treatment. The data are presented as mean ± SD (n=3, p=0.003). (F) ATP turnover of control (NC) and siRNA groups were calculated by the OCR of baseline minus oligomycin treatment. The data are presented as mean ± SD (n=3).

To investigate the effect of Lon knockdown on cellular bioenergetics of bladder cancer cells, we first assessed the real-time oxidative phosphorylation (OXPHOS) by measuring the cellular oxygen consumption rates (OCR) and glycolysis by assaying the extracellular acidification rate (ECAR) in UM-UC-3 and ScaBER cells with extracellular flux analyzer. OCR and ECAR are indicators of mitochondrial respiration and lactic acid production via aerobic glycolysis in cells, respectively. OCR for control and Lon down-regulated UM-UC-3 cells throughout bioenergetics analysis are shown in Figure 5A. Overall, Lon depletion using siRNA resulted in a marked reduction of the total mitochondrial respiratory capacity compared to the control cells as assessed by AUC (Figure 5B). Reduced basal OCR and ECAR in Lon down-regulated cells are showed in Figure 5C. Down-regulation of Lon significantly reduced basal OCR compared to that in control cells (Figure 5D), which was blocked by the ATP synthase inhibitor oligomycin, suggesting that Lon knockdown leads to lower energy production from OXPHOS of bladder cancer cells (Figure 5A), indicating the additional ATP requirement must be applied from glycolysis via increasing its activity. In addition, injecting the uncoupler FCCP increased OCR both in Lon siRNA cells and control cells, but Lon siRNA cells showed less OCR increase (Figure 5A). Finally, addition of ETC inhibitors rotenone and antimycin A blocked all electron transfer through complex I and complex III, led to a dramatically decrease in OCR in both control and Lon siRNA cells, and the OCR under the line of rotenone/

antimycin A-treated was contributed to capacity for ROS production and non-mitochondrial O_2_ consumption (Figure 5A). To further evaluate the difference of OCR variation between Lon siRNA and control cells, we assessed the mitochondrial function parameters by analyzing the OCR data at time point1-12, and we found markedly decrease of basal respiratory as well as ECAR at basal condition in Lon knockdown cells (p=0.002, Figure 5D), which indicated that Lon may play a crucial role in regulating cellular energy metabolism. Moreover, maximal respiratory and ATP production were also decreased due to Lon down-regulation (p=0.003 and p=0.002, Figure 5E and 5F), and which suggest that Lon may interact with the complexes of ETC to regulate the complexes activities given that the molecular chaperone properties of Lon protease. Further work need to be done to uncover the molecular mechanism of Lon regulating the bioenergetics of bladder cancer cell. Together, these data suggest that Lon may act as a vital modulator of cellular bioenergetics in bladder cancer cells.

### Lon expression in TMA and its correlation with clinicopathological parameters of bladder cancer

**Figure 6:**
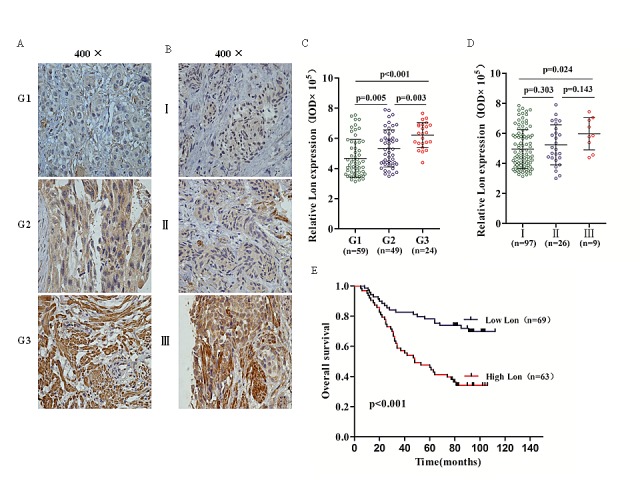
Immunohistochemical (IHC) staining of Lon protein in bladder cancer tissues and Kaplan–Meier overall survival curve of bladder cancer patients according to Lon expression (A & B) Representative Lon IHC staining photomicrographs (400×) of Grade 1, Grade 2 and Grade 3 bladder cancer tissues (A) and TNM stage I, II and III bladder cancer tissues (B). (C & D) Quantitative IHC results of Lon expression in different tumor grade (C) and different TNM stage (D). (E) The Kaplan-Meier overall survival curve of bladder cancer patients (n=132) according to Lon protein expression (p<0.001).

To further assess whether Lon protein expression is elevated in the tumor tissues and determine its association with clinical and pathologic parameters of bladder cancer patients, we performed immunohistochemical staining (IHC) in TMA containing 132 archived paraffin-embedded bladder cancer samples. As shown in Figure 6A and 6B, most of the bladder cancer cells were positive stained, though sporadic negative staining on these cells was also observed and the intensity of the IHC staining was variable. The corresponding integrated optical density (IOD) value was quantified by Image-Pro Plus 6.0 and shown in Figure 6C and 6D. Analyzed by one-way ANOVA test, we found that Lon expression was lower in well differentiated (G1) bladder cancer tissues than moderately (G2) (p=0.005) and poorly differentiated (G3) (p<0.001) bladder cancer tissues (Figure 6C). And it also showed a dramatically increase of Lon expression in G3 group compared with that in G2 group (p<0.003) In addition, we found that no statistical significance of Lon expression in bladder cancer patients between TNM stage I and II stage (p=0.303, Figure 6D) and as well as stage II and stage III (p=0.143, Figure 6D);however, there is significant difference in Lon expression for BC patients in TNM stage I and III (p=0.024, Figure 6D). The association between Lon protein expression and clinicopathological characteristics of bladder cancer was analyzed by the chi-square test. As shown in Table 1, high expression of Lon was significantly associated with T stage (p=0.032), histological grade (p<0.001), and TNM stage (p=0.032). However, no significant relationship was found between Lon protein expression and variables such as gender and age.

**Table 1:** Association between Lon expression and various clinicopathological factors of bladder cancer patients

Variables	No. (n=132)	Lon protein expression		p value
		Low(n=69)	High(n=63)	
Gender Male Female	115 17	59 10	56 7	0.562
Age <70 ≥70	54 78	33 36	21 42	0.091
Grade G1 G2 G3	59 49 24	45 20 4	14 29 20	<0.001
T stage T1 T2 T3-T4	97 26 9	57 10 2	40 16 7	0.032
TNM stage I II III - IV	97 26 9	57 10 2	40 16 7	0.032

The χ2 test was used to analyze the relationship between Lon expression and clinicopathological characteristics. Statistical significance was set at p<0.05.

### Survival analysis

The prognostic value of Lon for overall survival in bladder cancer patients was evaluated by comparing the patients with high and low Lon expression. According to the Kaplan-Meier survival analysis, patients with high Lon expression had obviously lower overall survival rates than those with low Lon expression (Figure 6E, p<0.001). Univariate and multivariate analyses were conducted using Cox proportional hazards model to examine the impact of Lon expression and other clinicopathological parameters in bladder cancer patients. We found that Lon expression, gender, tumor pathological grade, T stage and TNM stage were significant prognostic factors in the univariate analysis (Table 2). And we also performed multivariate survival analysis by Cox proportional hazard model to estimate the effects of independent factors on survival. As shown in Table 2, multivariate analysis indicated that Lon expression was one of the independent prognostic factors, along with T and TNM stage.

**Table 2:** Univariate analysis and multivariate analysis identifies factors influencing the overall survival of bladder cancer patients^a^

Variables	Univariate analysis			Multivariate analysis		
	RR^b^	95%	p value	RR^b^	95%	p value
Lon	1.779	1.224-2.585	0.003	1.637	1.074-2.495	0.022
Age	0.973	0.670-1.412	0.885	0.967	0.654-1.431	0.867
Gender	0.039	0.004-0.421	0.008	0.000	0.000-1.32E151	0.943
Grade	1.438	1.120-1.846	0.004	1.033	0.746-1.430	0.846
T stage	1.780	1.294-2.449	<0.001	1.618	1.108-2.361	0.013
TNM stage	1.780	1.294-2.449	<0.001	1.618	1.108-2.361	0.013

a Cox's proportional hazards model was used to identify the factors that had a significant influence on survival. Statistical significance was set at p<0.05.

b RR, relative risk;

In order to further understand the significance of clinicopathological factors in predicting survival of bladder cancer patients, we analyzed the survival rate of grade (p=0.0007) and TNM stage (p<0.001) as shown in Figure 7A and 7F, respectively. Furthermore, in all three grade groups, high Lon expression patients exhibited significant lower survival rate in bladder cancer patients diagnosed with G3 (p=0.0388, Figure 7D); however, Lon expression plays no significant effect to the survival of bladder cancer patients with grade 1 and grade 2 (Figure 7B,=0.0809 and Figure 7C,=0.1906, respectively). Additionally, overall survival of the bladder cancer patients with high Lon expression in TNM Stage I and II was much worse (Figure 7F,=0.0359 and Figure 7G,=0.0110, respectively). While Lon expression did not predict outcome of TNM stage III bladder patients (p=0.1757, Figure 7H). Taken together, these findings indicate that Lon protein expression may be a useful independent prognostic factor for bladder cancer.

**Figure 7:**
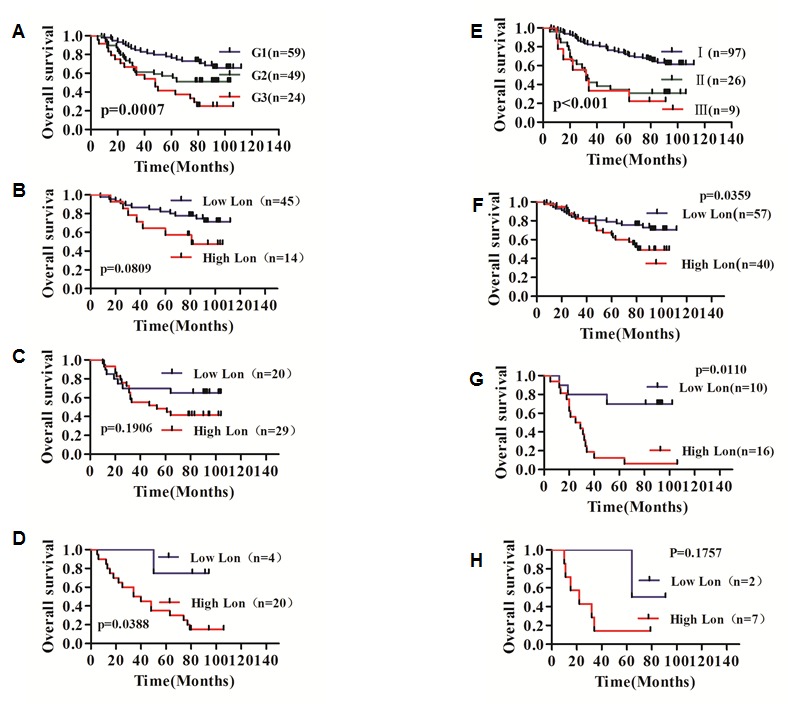
Kaplan–Meier overall survival analysis for bladder cancer patients in different grade and TNM stages carried out according to the expression status of Lon (A) Significant prognostic effect of cell differentiation (Grade) for BC patients survival (p=0.0007). (B, C & D) The effect of Lon expression on the survival of bladder cancer patients in the group of G1 (p=0.0809, B), G2 (p=0.1906, C) and G3 (p=0.0388, D), respectively. (E) TNM stage significantly influences BC patients survival (p<0.001). (F, G & H) The effect of Lon expression on the survival of bladder cancer patients in the group of TNM stage I (p=0.0359, F), stage II (p=0.0110, G) and stage III (p=0.0157, H), respectively.

## DISCUSSION

There is growing evidence that mitochondrial Lon protease plays a very active role in tumor progression. The mitochondrial respiratory chain is the major source of intracellular ROS, which may lead to the mitochondrial dysfunction by oxidation of mtDNA, lipids, as well as proteins. The protein quality control systems within mitochondrial matrix are mainly contributed to the repair or removal of the oxidized mitochondrial proteins to maintain the mitochondria function well.

Expression of rat Lon is increased in response to the oxidative or ER stress to regulate the important processes within mitochondrial such as assembly and/or degradation of cytochrome c oxidase (COX) [18]. The mtDNA encoded subunit II of COX was degraded rapidly and the steady-level of nuclear-encoded Subunit IV and V of COX were also reduced under ER stress. During hypoxia, hypoxia inducible factor 1 (HIF-1) activates the *LON* gene transcription which up-regulates Lon protease expression. Increased Lon protease degrades COX4-1 (isoform 1 of COX subunit 4) to facilitate the switch from COX4-1 to more efficient COX4-2 to challenge the low oxygen by enhancing mitochondrial respiration [17]. Thus, Lon protease mediated protein degradation can relieve the heavy load of abnormal mitochondrial proteins accumulation caused by ER tress and /or oxidative stress.

Our recent studies demonstrated that Lon expression was increased in cervical cancer tissues and Lon may serve as a potential therapeutic target in cervical cancer [33]. In human lung fibroblast cells, damaged protein aggregates rapidly in mitochondrial matrix when *LON* gene expression was blocked [34, 35]. In addition, transient down-regulation of Lon resulted in impaired mitochondrial function and apoptosis of human WI-38 VA-13 lung fibroblasts [21]. Human Lon protease is highly expressed in adipose tissue from patients with lipodystrophy, and the up-regulation is caused by nucleosidic reverse transcriptase inhibitor which induced ROS production [36]. Previous studies showed that Lon protease were up-regulated in human lung cancer cells and malignant B-cell lymphoma cells [21, 22]. Our recently published work shows that anti-cancer drug bortezomib can block the TFAM degradation and increase mtDNA copy numbers in cells with extremely low mtDNA by inhibiting Lon protease activity [22].In human oral cancer cell OEC-M1 and FADU, over-expression of Lon protease resulted in the enhancing of mitochondrial ROS generation, which is related to Lon-mediated up-regulation of a mitochondrial Fe-S protein in complex I of electron transport chain (NDUFS8) [28]. We speculate that Lon may involve in bladder cancer progression, which could serve as a novel diagnostic and /or prognostic biomarker for BC, as well as signs of bladder cancer progression and therapeutic target for the treatment of BC. To confirm our hypothesis, we first analyzed expression profile of Lon in bladder cancer cell lines, and found that Lon was steady expressed in these bladder cancer cell lines both in protein and mRNA level. In this study, we also showed that both the Lon mRNA and protein expression levels were significantly higher in bladder cancer tissues than that in adjacent normal tissues.

Maintenance of energy homeostasis is critical for cell proliferation, survival, differentiation and apoptosis. To investigate the role of Lon in bladder cancer progression, we down-regulated the Lon expression in bladder cancer cells ScaBER and UM-UC-3 using siRNA, and we found that the down-regulation of endogenous Lon reduced the cell proliferation of ScaBER and UM-UC-3 cells, and decreased the cellular energy metabolism. Down-regulation of Lon protease further leads to the impairment to the assembly and/or function of mitochondrial respiratory chain complexes and inhibits the mitochondrial ROS production, and decreased ROS level inhibits cancer cell proliferation, cell survival. In addition, the growth inhibition of bladder cancer cells could be partially attributed to the dysfunction of protein quality control systems which causes cancer cells failed to prevent mitochondrial proteotoxicity due to deficiency of Lon protease and the decreased chaperone activity of Lon, which resulting in the pernicious accumulation of misfolded, unassembled and/or oxidatively damaged proteins [21,37].

Over-expression of heat shock protein (HSP) family member molecular chaperones as well as 26S proteasome powered by ATP plays critical roles in assisting various cancer cell growth and survival, and these chaperon proteins may serve as nononcogenic protein target for cancer therapy[38,39,40]. The ATP-dependent Lon protease has similar function within mitochondrial matrix to facilitate cells adapting to various proteotoxic stresses related to oncogenesis. We proposed that Lon might also be a potential anticancer drug target. To better understand the roles of Lon protease as an anticancer drug target, further investigations are required to clarify the molecular mechanisms. Our data indicated that knockdown the Lon enhances the sensitivity of bladder cancer cells UM-UC-3 to doxorubicin by facilitating caspase-dependent apoptosis. Moreover, down-regulation of Lon decreased mitochondrial ROS production and blocks c-Jun N-terminal kinases (JNK) activation, which suggest that down-regulation of Lon reduce ROS production in bladder cancer cells, and may cause the inhibition of bladder cancer cell proliferation through suppression JNK phosphorylation in ROS-induced MAPK pathway. Altogether, these data suggest that Lon may play an important role in bladder cancer progression via involving in chemoresistance. Based on our results, we speculate that inhibitors targeting Lon may be a potential strategy to bladder cancer treatment.

To further investigate whether Lon expression might be associated with the progression of bladder cancer, the Lon protein expression levels and the clinic pathologic characteristics of 132 patients with bladder cancer were analyzed by immunohistochemistry. Our data indicate that high Lon expression is significantly correlated with tumor grade, T stage and TNM stage. Kaplan–Meier survival analysis showed that high Lon expression is negatively correlated with the overall survival of patients with bladder cancer. More importantly, further analysis using the Cox regression model confirmed that the Lon expression was an independent factor in predicting overall survival time for bladder cancer patients. These results suggest that Lon might serve as a valuable prognostic biomarker for bladder cancer patients after surgery and as a potential therapeutic target in the treatment of bladder cancer. Our findings show for the first time that the expression level of Lon was up-regulated in bladder cancerous tissues, and reveal an essential role for Lon protease in the tumorigenesis of bladder cancer.

In conclusion, our study suggests that Lon supports bladder tumorgenesis and may be a novel drug target and prognostic marker of bladder cancer. These findings also provide us a rationale for elucidating the role of ATP-dependent Lon protease in other types of cancer and for further attempts to block cancer cell proliferation and increases chemosensitivity by inhibiting Lon protease activity and decrease Lon expression. Finally, Lon may also be a potential molecular marker for preoperative chemotherapy treatment as well as for prediction of the effectiveness of chemotherapy.

## MATERIALS AND METHODS

### Chemicals and antibodies

Doxorubicin, oligomycin, carbonylcyanide-p-trifluoromethoxyphenylhydrazone (FCCP), antimycin A, rotenone were obtained from Sigma (St. Louis, MO). MitoSox^TM^ Red was purchased from molecular probes (Eugene, OR). Horseradish peroxidase (HRP)-conjugated anti-rabbit, anti-mouse immunoglobulin G, reactive oxygen species assay kit (DCFH-DA) and cell counting kit-8 were obtained from Beyotime (Haimen, Jiangshu, China). Trypan blue was obtained from Life Technologies (Carlsbad, CA). ECL reagent was obtained from Thermo scientific (Waltham, MA).

The polyclonal antibody against Lon synthetic peptide (CRRPQDKDAKGDKDG) was raised in rabbit and affinity purified by GenScript (Nanjing, Jiangshu, China). The monoclonal antibody against β-actin was from Abmart (Shanghai, China). The polyclonal antibody against TFAM was from Proteintech (Wuhan, China). Antibodies recognizing PARP, Caspase-3, p53, JNK, phospho-JNK (Thr183/Tyr185), AKT and phospho-AKT were from Cell Signaling Technology (Beverly,MA).

### Cell lines and cell culture

The human bladder cancer cell line UM-UC-3, SCaBER, SW780, J82 and T24 were purchased from American Type Culture Collection (ATCC) (Manassas, VA) and cultured in Dulbecco's Modified Eagle's Medium (DMEM) (Life Technologies, Grand Island, NY) supplemented with 10% fetal bovine serum (FBS) (Life Technologies, Grand Island, NY) and antibiotics (100 U/ml penicillin and streptomycin) at 37°C in a humidified incubator with 5% CO_2_.

### Patients and samples

Samples from 45 tumors (each sample including paired normal and tumor tissues from the same patient) were obtained from untreated patients who underwent surgical treatment for bladder cancer at Department of Urology of the Second Affiliated Hospital of Wenzhou Medical University, Wenzhou, China between 2006 and 2013. The patients age ranged from 35 to 86 years (mean 68.5 years). All the tissue samples were divided into two parts. One part was embedded in paraffin and processed for routine histopathological examination, and the remainder tissue was immediately shock frozen in liquid nitrogen and then stored at −80 ºC for later studies. All the patients were clinically and pathologically confirmed to be bladder cancer. The tumor stage and grade were defined according to the American Joint Committee on Cancer (AJCC) /Union InternationaleContre Cancer (UICC) and WHO-classification. This study was approved by the Board and Ethical Committee of the Second Affiliated Hospital of Wenzhou Medical University. Written informed consent was obtained from all patients participated in this study.

### RNAi and transfection

RNAi-mediated gene knockdown was done with the 19-nucleotide targets using siRNAs (GenePharma, Shanghai, China) as follows: Lon siRNA sense: 5′-GGAGCAGCUAAAGAUCAUCTT-3′ Lon siRNA antisense: 5′-GAUGAUCUUUAGCUGCUCC TG-3′ Control siRNA sense: 5′-UUCUCCGAACGUGUCACGUTT-3′ Control siRNA antisense: 5′- ACGUGACACGUUCGGAGAATT-3′ UM-UC-3 and SCaBER cells (2×10^5^) were transfected with a final concentration of 100 nmol/L siRNAs using Lipofectamine 2000 (Life Technologies, Carlsbad, CA) according to manufacturer's instructions.

### Cell proliferation and viability analysis

UM-UC-3 and SCaBER cells transfected with control and Lon siRNA, and cells were harvested and resuspended in DMEM medium 24 h post transfection. Cells were seeded into a 6-well plate at a density of 4×10^3^ cells per well and incubated overnight. The viability of ScaBER and UM-UC-3cells was determined by counting living and dead cells by Trypan blue dye (0.05% solution) exclusion method using a hemocytometer and cell counting kit-8 (Beyotime, Jiangshu, China), respectively. For MTT assay, UM-UC-3 and ScaBER cells were seeded into 96-well plates at a density of 1×10^5^ cells/well and incubated overnight. Cells were treated with a concentration gradient of doxorubicin. After treatment for12 h, cells were stained with MTT reagent (5 mg/ml) (Beyotime, Jiangshu, China). After 4 h incubation with MTT reagent, the crystals produced were then dissolved with DMSO. Once the crystals were dissolved completely, 100 μL of solution were transferred to 96-well plate and measured at 570 nm by plate reader (Thermo scientific, USA)

### Reactive oxygen species (ROS) determination

Intracellular ROS was measured by using the fluorescence probe DCFH-DA according to manufacturer's protocol (Molecular Probes, Eugene, OR). Briefly, cells were incubated with 10 mM DCFH-DA at 37 ºC for 20 min, followed by washing three times with medium to remove extracellular DCFH-DA and left appropriate volume of Serum free medium for flow cytometry analysis. Next, mitochondrial superoxide production was determined using MitoSOX^TM^ Red regent (5μM) according to the manufacturer's instruction. Forty-eight hour post transfection, 3 ×10^5^ cells (control or Lon siRNA UM-UC-3 cells) were seeded into 6 well-plates and incubated at 37 ºC, 5% CO for 24 h. Then, live-cell imagers were taken with fluorescence microscopy.

### Doxorubicin-sensitivity assay

UM-UC-3 cells were transfected with Lon and negative control siRNA, and resuspended in DMEM medium 24 h post transfection. Cells were then seeded into 6-well plate at a density of 4×10^5^ cells/well and incubated at 37 ºC, 5% CO_2_ for 24 h. Next, cells were treated with a concentration gradient of doxorubicin (0, 0.5, 1, 2, 4μg/μL). Then cells were collected and subjected to western blotting with indicated antibodies

### Western blot analysis

Each sample equivalent of 20 μg total protein was separated by 10% SDS-PAGE gels followed by electrophoretic transfer onto nitrocellulose membrane (Bio-Rad, Hercules, CA) in Tris-glycine buffer. Blots were blocked at room temperature for 1.5 h in blocking buffer (3% non-fat milk in TBST) on a shaker, and then incubated with primary antibodies specific to Lon and β-actin overnight at 4 ºC, respectively. The membrane was washed in TBST for 3×10 min and then incubated with horseradish peroxidase (HRP)-conjugated anti-rabbit and anti-mouse immunoglobulin G at room temperature for 1h while gentle agitating,respectively. Immunreactive proteins were visualized using ECL reagent according to the manufacturer`s protocol (Thermo Scientific, Rockford, IL). The optical density was quantified by performing the National Institute of Health Image J software.

### Tissue microarrays (TMA) and immunohistochemical (IHC) staining

Bladder cancer TMA containing a total of 132 formalin-fixed, paraffin-embedded tissue sections (4 μm thickness) were constructed as described by Nocito *et al*. [41]. IHC staining of TMA was performed according to standard protocol. Briefly, the sections on TMA were deparaffinized in xylene and rehydrated with a gradient ethanol concentrations, the endogenous peroxidase activity was quenched, non-specific staining was blocked by 5% normal goat serum, and followed by incubation of TMA slides with ant-Lon antibody and also without primary antibody (using PBS instead of anti-Lon) as a negative control overnight at 4°C. The slides were then incubated with biotin-labeled goat anti-rabbit Ig G and further incubated with streptavidin peroxidase solution (SABC kit, Boster, Wuhan, China). The staining was visualized by reaction with 3, 3-di-aminobenzidine (Boster, Wuhan, China) in PBS with 0.05% H_2_O_2_ for 5 min at room temperature and followed by counterstaining with hematoxylin.

### Assessment of IHC staining

The TMA were reviewed and scored independently by two pathologists who had no prior knowledge of the clinicopathological features of the specimens on the TMA. For IHC quantification, three random representative 400× microscopic fields per section were photographed using a standard Nikon Light Microscope. Then using imaging analysis software Image-Pro Plus 6.0 (IPP) (Media Cybernetics, Rockville, MD) for digital photographs analysis by calculation of integrated optical density (IOD) referencing the method introduced previously [42]. IOD, the integral calculus of the stained area times the intensity of staining in each pixel in the area, indicates the total amount of staining material in that area. Lon protein expression levels in the TMA were evaluated by scanning the entire tissue microarrays.

The IHC staining of Lon was assessed according to the immune-reactive score (IRS) as described previously [43, 44], and adjusted slightly, which evaluated both the percentage of positive cells and the staining intensity. The percentage of positive cells was scored as 1 (≤10%), 2 (10-50%), 3 (50-80%), 4 (≥80%)[45]; staining intensity was graded as 0 (negative), 1 (weak), 2 (moderate), and 3 (strong). The two scores were multiplied and the IRS (values from 0 to 12) was determined as “Low” and “High” corresponding to IRS values of ≤ 6 and>6, respectively.

### RNA preparation and quantitative real-time PCR

Total RNA was extracted from normal and cancerous tissues of bladder cancer patients using Trizol reagent (Life Technologies, Carlsbad, CA) according to the manufacturer`s instructions. Total RNA (2μg) was used to synthesis first-strand cDNA by reverse transcription using PrimeScript^TM^ RT reagent Kit with gDNA Eraser (Takara, Dalian, China) according to the manufacturer`s instructions. The RT reaction was subsequently used as a template for real-time PCR. Real-time PCR assays were performed using 2μL cDNA /20μL reaction volume on a CFX connect^TM^ real-time system (Bio-Rad) using SYBR Green according to the manufacturer's protocol. Primer sequences are as follows: forward: 5`-GCTGCTGAGAAGGAAAGTTCG-3`; reverse: 5`-CGTGTGGTAGATTTCATCCAG G-3`. Thermal cycling was performed using the following parameters: 95 ºC for 10 min, 45 cycles of denaturation at 95 ºC for 10 s and extension at 60 ºC for 30 s. The threshold cycle number (CT) was recorded for each reaction. The CT value of Lon was normalized to that of β-actin. Each sample was analyzed in triplicate and repeated 3 times.

### Oxidative phosphorylation and glycolysis Assay

The intact cellular oxygen consumption rate (OCR) and extracellular acidification rate (ECAR) were measured in real time using the Seahorse XF96 Extracellular Flux Analyser (Seahorse Bioscience, North Billerica, MA, USA) as previous described [33]. Briefly, 1.0 ×10^4^ of ScaBER or UM-UC-3 cells were seeded into 96 well Seahorse microplates in 80 μL of growth medium and incubated at 37 ºC in 5% CO2 for 24 h and the the calibrator plate was equilibrated in a non-CO_2_ incubator overnight. Before starting the test, cells were washed twice with assay running media (unbuffered DMEM, 25mM glucose, 1mM glutamine, 1mM sodium pyruvate) and equilibrated in a non-CO_2_ incubator. Once the probe calibration was completed, the probe plate was replaced by the cell plate. The protocol was optimized and gave the measurement of oxygen consumption rate (OCR) and extracellular acidification rate (ECAR) simultaneously. Totally, the assay protocol incorporated four compounds injection which could be applied to modulate mitochondrial function to determine some of the mitochondrial parameters, including basal respiration, ATP production and maximal respiration. The analyzer plotted the value of OCR and the corresponding ECAR followed by injection of the compounds sequentially as follows: oligomycin (1μM), an inhibitor of ATP synthase which leads to exhibit maximal glycolysis metabolism; followed by exposure of carbonylcyanide p-trifluoromethoxyphenylhydrazone (FCCP) (1μM), the uncoupler of ETC and OXPHOS which induces the peak oxygen consumption to evaluate the crest oxidative metabolism indirectly; by addition of ETC inhibitor rotenone and the complex III inhibitor antimycin A to uncover the part of non-mitochondrial respiration at a final concentration of 1μM. The control (NC) and Lon siRNA groups include three replicates and the results were obtained by performing three independent experiments. Once the assay was completed, BCA Protein Assay Kit (Thermo scientific, USA) was performed to determine the protein concentration to normalize the value of OCR and ECAR according to manufacturer's protocol.

### Statistical Analyzes

Expression level of Lon was quantified relative to β-actin, a loading control protein. The Lon expression level of normal tissues was defined as 100%. The expression levels of cancer tissues were a relative expression level to normal tissues. All statistical analyses were performed with the SPSS 13.0 statistical software package (SPSS Standard version 16.0, SPSS Inc., Chicago, IL). Wilcoxon signed-rank test was used to analyze the significance of protein and mRNA expression in normal and cancer tissues of bladder. The χ^2^ test and one-way ANOVA analysis were performed to evaluate the relationship between the clinicopathological features and Lon expression in the IHC results. Kaplan–Meier survival analysis was used to evaluate patient's prognosis. The overall survival rate of the bladder cancer patients was obtained using the life table method. A univariate analysis was plotted using the Kaplan–Meier method. Cox regression analysis was used to evaluate the relationship between molecular parameters and the clinicopathological parameters; p≤0.05 was considered to be statistically significant.

## References

[1] Venkatesh S, Lee J, Singh K, Lee I, Suzuki CK (2012). Multitasking in the mitochondrion by the ATPdependent Lon protease. Biochim Biophys Acta.

[2] Lee I, Suzuki CK (2008). Functional mechanics of the ATP-dependent Lon protease-lessons from endogenous protein and synthetic peptide substrates. Biochim Biophys Acta.

[3] Jemal A, Siegel R, Xu J, Ward E (2010). Cancer statistics, 2010. CA Cancer J Clin.

[4] Clavel J, Cordier S, Boccon-Gibod L, Hemon D (1989). Tobacco and bladder cancer in males: increased risk for inhalers and smokers of black tobacco. Int J Cancer.

[5] Botteman MF, Pashos CL, Redaelli A, Laskin B, Hauser R (2003). The health economics of bladder cancer: a comprehensive review of the published literature. Pharmacoeconomics.

[6] Avritscher EB, Cooksley CD, Grossman HB, Sabichi AL, Hamblin L, Dinney CP, Elting LS (2006). Clinical model of lifetime cost of treating bladder cancer and associated complications. Urology.

[7] Dey P (2004). Urinary markers of bladder carcinoma. Clin Chim Acta.

[8] Tsilibaris V, Maenhaut-Michel G, Van Melderen L (2006). Biological roles of the Lon ATP-dependent protease. Res Microbiol.

[9] Lee I, Suzuki CK (2008). Functional mechanics of the ATP-dependent Lon protease-lessons from endogenous protein and synthetic peptide substrates. Biochim Biophys Acta.

[10] Bogenhagen DF, Rousseau D, Burke S (2008). The layered structure of human mitochondrial DNA nucleoids. J Biol Chem.

[11] Lu B, Liu T, Crosby JA, Thomas-Wohlever J, Lee I, Suzuki CK (2003). The ATP-dependent Lon protease of Mus musculus is a DNA-binding protein that is functionally conserved between yeast and mammals. Gene.

[12] Pinti M, Gibellini L, De Biasi S, Nasi M, Roat E, O'Connor JE, Cossarizza A (2011). Functional characterization of the promoter of the human Lon protease gene. Mitochondrion.

[13] Fu GK, Markovitz DM (1998). The human LON protease binds to mitochondrial promoters in a single-stranded, site-specific, strand-specific manner. Biochemistry.

[14] Liu T, Lu B, Lee I, Ondrovicova G, Kutejova E, Suzuki CK (2004). DNA and RNA binding by the mitochondrial lon protease is regulated by nucleotide and protein substrate. J Biol Chem.

[15] Bota DA, Davies KJ (2002). Lon protease preferentially degrades oxidized mitochondrial aconitase by an ATP-stimulated mechanism. Nat Cell Biol.

[16] Granot Z, Kobiler O, Melamed-Book N, Eimerl S, Bahat A, Lu B, Braun S, Maurizi MR, Suzuki CK, Oppenheim AB, Orly J (2007). Turnover of mitochondrial steroidogenic acute regulatory (StAR) protein by Lon protease: the unexpected effect of proteasome inhibitors. Mol Endocrinol.

[17] Fukuda R, Zhang H, Kim JW, Shimoda L, Dang CV, Semenza GL (2007). HIF-1 regulates cytochrome oxidase subunits to optimize efficiency of respiration in hypoxic cells. Cell.

[18] Hori O, Ichinoda F, Tamatani T, Tamatani T, Yamaguchi A, Sato N, Ozawa K, Kitao Y, Miyazaki M, Harding HP, Ron D, Tohyama M, Stern DM, Ogawa S (2002). Transmission of cell stress from endoplasmic reticulum to mitochondria: enhanced expression of Lon protease. J Cell Biol.

[19] Goard CA, Schimmer AD (2013). Mitochondrial matrix proteases as novel therapeutic targets in malignancy. Oncogene.

[20] Bulteau A, Bayot A (2011). Mitochondrial protease and cancer. Biochim. Biophys.Acta.

[21] Bota DA, Ngo JK, Davies KJ (2005). Down-regulation of the human Lon protease impairs mitochondrial structure and function and causes cell death. Free Radic Biol Med.

[22] Bernstein SH, Venkatesh S, Li M, Lee J, Lu B, Hilchey S, Morse KM, Metcalfe HM, Andreeff M, Brookes PS, Suzuki CK (2012). The mitochondrial ATP-dependent Lon protease: a novel target in lymphoma death mediated by the synthetic triterpenoid CDDO and its derivatives. Blood.

[23] Xue X, Zhu YF, Mao JP (2007). Effect of RNA interference for Lon gene silencing on growth and apoptosis of human breast cancer MCF7 cells. Nan Fang Yi Ke Da Xue Xue Bao.

[24] Wang HM, Cheng KC, Lin CJ, Hsu SW, Fang WC, Hsu TF, Chiu CC, Chang HW, Hsu CH, Lee YL (2010). Obtusilactone A and (−)-sesamin induce apoptosis in human lung cancer cells by inhibiting mitochondrial Lon protease and activating DNA damage checkpoints. Cancer Sci.

[25] Zhao Y, Wang ZB, Xu JX (2003). Effect of cytochrome c on the generation and elimination of O2*-and H2O2 in mitochondria. J Biol Chem.

[26] Kim EK, Choi EJ (2010). Pathological roles of MAPK signaling pathways in human diseases. Biochim Biophys Acta.

[27] Luciakova K, Sokolikova B, Chloupkova M, Nelson BD (1999). Enhanced mitochondrial biogenesis is associated with increased expression of the mitochondrial ATP-dependent Lon protease. FEBS Lett.

[28] Cheng C-W, Kuo C-Y, Fan C-C, Fang W-C, Jiang SS, Lo Y-K, Wang T-Y, Kao M-C, Lee AY-L (2013). Overexpression of Lon contributes to survival and aggressive phenotype of cancer cells through mitochondrial complex I-mediated generation of reactive oxygen species. Cell death and disease.

[29] Lu B, Lee J, Nie XB, Li M, Morozov YI, Venkatesh S, Bogenhagen DF, Temiakov D, Suzuki CK (2013). Phosphorylation of human TFAM in mitochondria impairs DNA binding and promotes degradation by the AAA+ Lon protease. Molecular Cell.

[30] Qin ZK, Yang JA, Ye YL, Zhang X, Xu LH, Zhou FJ, Han H, Liu ZW, Song LB, MS Zeng (2009). Expression of Bmi-1 is a prognostic marker in bladder cancer. BMC Cancer.

[31] Raitanen MP, Marttila T, Kaasinen E, Rintala E, Aine R, Tammela TL (2000). Sensitivity of human complement factor H related protein (BTA stat) test and voided urine cytology in the diagnosis of bladder cancer. J Urol.

[32] Budman LI, Kassouf W, Steinberg JR (2008). Biomarkers for detection and surveillance of bladder cancer. Can Urol Assoc J.

[33] Nie XB, Li M, Lu B, Zhang YX, Lan LH, Chen L, Lu JX (2013). Down-regulating overexpressed human Lon in cervical cancer suppresses cell proliferation and bioenergetics. PLOS ONE.

[34] Ngo JK, Pomatto LC, Bota DA, Koop AL, Davies KJ (2011). Impairment of Lon-Induced Protection Against the Accumulation of Oxidized Proteins in Senescent Wi-38 Fibroblasts. J Gerontol A Biol Sci Med Sci.

[35] Ngo JK, Pomatto LC, Davies KJ (2013). Upregulation of the mitochondrial Lon Protease allows adaptation to acute oxidative stress but dysregulation is associated with chronic stress, disease, and aging. Redox Biol.

[36] Pinti M, Gibellini L, Guaraldi G, Orlando G, Gant TW, Morselli E, Nasi M, Salomoni P, Mussini C, Cossarizza A (2010). Upregulation of nuclear-encoded mitochondrial LON protease in HAART-treated HIV-positive patients with lipodystrophy: implications for the pathogenesis of the disease. AIDS.

[37] Erjavec N, Bayot A, Gareil M, Camougrand N, Nystrom T, Friguet B, Bulteau AL (2013). Deletion of the mitochondrial Pim1/Lon protease in yeast results in accelerated aging and impairment of the proteasome. Free Radic Biol Med.

[38] Dai C, Whitesell L, Rogers AB, Lindquist S (2007). Heat shock factor 1 is a powerful multifaceted modifier of carcinogenesis. Cell.

[39] Leu JI, Pimkina J, Frank A, Murphy ME, George DL (2009). A small molecule inhibitor of inducible heat shock protein 70. Mol Cell.

[40] Trepel J, Mollapour M, Giaccone G, Neckers L (2010). Targeting the dynamic HSP90 complex in cancer. Nat Rev Cancer.

[41] Nocito A, Bubendorf L, Tinner EM, Süess K, Wagner U, Forster T, Kononen J, Fijan A, Bruderer J, Schmid U, Ackermann D, Maurer R, Alund G, Knönagel H, Rist M, Anabitarte M (2001). Microarrays of bladder cancer tissue are highly representative of proliferation index and histological grade. J. Pathol.

[42] Wang CJ, Zhou ZG, Holmqvist A, Zhang H, Li Y, Adell G, Sun XF (2009). Survivin expression quantified by Image Pro-Plus compared with visual assessment. Applied immunohistochemistry & molecular morphology : AIMM / official publication of the Society for Applied Immunohistochemistry.

[43] Remmele W, Stegner HE (1987). Recommendation for uniform definition of an immunoreactive score (IRS) for immunohistochemical estrogen receptor detection (ER-ICA) in breast cancer tissue. Pathologe.

[44] Cheng AN, Jiang SS, Fan CC, Lo YK, Kuo CY, Chen CH, Liu YL, Lee CC, Chen WS, Huang TS, Wang TY, Lee AY (2013). Increased Cdc7 expression is a marker of oral squamous cell carcinoma and overexpression of Cdc7 contributes to the resistance to DNA-damaging agents. Cancer letters.

[45] Bolander Å, Agnarsdóttir M, Strömberg S, Ponten F, Hesselius P, mathias Uhlenand M, Bergqvist M (2008). The protein expression of TRP-1 and galectin-1 in cutaneous malignant melanomas. Cancer genomics & proteomics.

